# Sustainable, greenhouse gas derived fermented protein in canine diets—a pilot study

**DOI:** 10.3389/fvets.2024.1477182

**Published:** 2025-01-10

**Authors:** Ravindra Babu, Sreedevi Padmanabhan, Ravikumar Ganesan, Ezhil Subbian, Thi Thu Hao Van, Rajaraman Eri

**Affiliations:** ^1^String Bio Private Limited, Bengaluru, Karnataka, India; ^2^School of Science, STEM College, RMIT University, Melbourne, VIC, Australia

**Keywords:** fermented protein, greenhouse gas, microbiome, canine diet, palatable

## Abstract

Sustainability concerns have increased consumer demand for non-animal-derived proteins and the search for novel, alternative protein sources. The nutritional sustainability of the food system without compromising the nutrient quality, composition, digestibility and consumption is pivotal. As with farmed livestock, it is imperative to ensure the well-being and food security of companion animals and to develop sustainable and affordable pet foods. The current pilot study was conducted to determine the effect of greenhouse gas-derived novel, fermented protein ingredient in beagle dogs. The greenhouse gas-derived fermented protein is an alternative protein ingredient with optimal nutritional factors and provides traceability, significantly optimizes the use of land and water, and provides sustainability to the feed value chain of canine diets. Three experimental groups including control, 5 and 10% inclusion of high protein ingredients were included in the study and the results suggest that the fermented protein is palatable and acceptable at 5 and 10% inclusions in the diets of dogs. The present study shows no significant difference in general alertness, clinical symptoms, water consumption and social behavior of dogs between 5 and 10% fermented protein inclusion in canine diets. The diversity of the bacterial community did not change after supplementation with the tested protein source in dogs. Only a few bacterial genera differed significantly in relative abundance between the experimental groups. Feed consumption, faecal scoring and the microbiome data results of this pilot study on the use of novel, methane gas derived, bacterial SCP as a protein ingredient in the canine diets, would pave way for more and more inclusion of such novel alternative protein sources in the pet food industry.

## Introduction

The global pet food market is projected to grow from 115.50 billion USD in 2022 to 163.70 billion USD by 2029 (1). The increasing trend for pet ownership, rising urbanization and pet humanization are factors for the pet owners to opt also for nutritious and quality food for their pets and act as major drivers in the petfood market. Proteins in pet diets sourced from animal origin are posing threats on the sustainability factor. Hence adoption of sustainable practices of developing feeds less reliant on non-renewable sources would significantly strike the right balance between nutritional, ecological, social and economic aspects.

Protein is the most expensive, indispensable macronutrient in pet foods. National Research Council (NRC) (73) provides a recommended allowance of 10 and 20% crude protein for adult dogs (2) whereas the recommendations made by AAFCO for adult dogs is 18% crude protein (3). The ideal amino acid profile for dog nutrition is provided by Baker and Maulden (4). The advantages of high protein, low carbohydrate foods elicit lower glycemic index which can benefit dogs with insulin resistance and diabetes (5, 6). The protein content of the diet is positively associated with food selection in dogs (7). Studies have shown that pet foods with a higher protein content (103 g/1000 kcal) in addition to higher fiber content, decrease voluntary intake, increase the amount and rate of weight loss, and increase fat mass loss during weight loss in dogs (8, 9). Dog foods containing high protein and low energy maintain muscle mass during weight loss (10, 11). Additionally, high-protein diets can be beneficial for endurance exercise in dogs. Sled dogs fed a diet consisting of 35% of energy from protein had higher plasma volume than dogs fed a diet with 18% of energy from protein (12). The 18% protein diet also resulted in decreased maximal oxygen uptake (VO2 max) and greater rate of soft-tissue injuries.

Considering the critical role of protein in pet foods, and in response to consumer demand, sourcing constraints and sustainability concerns, research for novel protein sources have emerged as an important trend in the pet food industry. Dried whole-cell yeast (*S. cerevisiae*) is an alternative to conventional animal-derived protein sources that aligns with this trend and has been shown to have beneficial health effects in several animal species, including the modulation of the colonic microbiota in dogs (13–16). Insect based proteins are also tested in dogs wherein the diets were shown to alter the gut microbiota slightly (17). Bacterial based protein ingredients which are produced under controlled conditions, and which are scalable are considered viable alternative source to circumvent the problems of protein shortage. Existing pet foods are rich in ingredients of animal origin and are associated with drawbacks such as higher greenhouse gas emissions, land and water use. A recent study estimated that pet food, specifically dry diets from the U.S., could account for up to 2.9% of global CO2 equivalent emissions and up to 1.2% of agricultural land use. As greenhouse gas, methane contributes to the global warming potential (GWP_20_) 84 times that of carbon di oxide (18, 19), methane removal technologies have gained significant attention (20, 21) and is also considered as a cost-efficient carbon and energy source from the biomanufacturing standpoint (22). Fermented proteins offer several advantages over animal and plant proteins such as low carbon footprint, low reliance on land, water, and seasonal variations coupled with a balanced amino acid and nutritional profile. The current study provides support for the acceptability and digestibility of dog diets containing such greenhouse gas derived microbial fermented protein as a sustainable alternative protein source with an ideal amino acid profile and is palatable.

## Materials and methods

### Animals, facilities, and experimental design

Clinically healthy, adult beagle dogs, of both sexes, between 12 and 20 kg of bodyweight were enrolled in the study. Beagles were utilized in the study due to their uniform sizes, excellent temperament and physiology suited to studies in controlled environments. The standard housing conditions required for canine studies such as provision of minimum 2–4 m^2^ space for the dogs allowing for free movement, non-slip flooring, soft bedding materials (straw). The animals had constant human interaction in addition to additional props (chew toys) for environmental enrichment. Adequate environmental conditions (temperature, humidity, ventilation, lighting) were maintained as per standard.

### Dry pet food preparation as kibbles

The dry feed (kibbles) for dogs used in this study were custom manufactured by a pet food manufacturer (Taiyo Group, Chennai, India) and consisted of three different formulations using fermented single cell proteins (SCP) of bacterial origin with three different inclusion levels (0, 5, and 10%) stored at room temperature in sealed packages. Each sample is derived from the same lot of production, using uniform production parameters. Based on the inclusion levels of fermented proteins, each formulation also comprises varying percentages of different cereals and grain byproducts and micronutrients as mentioned in Table 1 as control, test 1 (fermented protein 5% inclusion), test 2 (fermented protein 10% inclusion) respectively. The fermented protein was produced by the continuous aerobic fermentation process using a patented proprietary fermentation process of String Bio Pvt. Ltd., India within its String Integrated Methane Platform, SIMP™ technology, as described in Subbian et al. (23). The same extrusion technique was used for the production of all of the formulations, and processing was carried out under the same conditions. The protein content of the different formulations was maintained at around 24%.

**Table 1:** Ingredient composition (%) of dry dog feeds used in the study.

Ingredients	Control	Test 1 (fermented protein 5% inclusion)	Test 2 (fermented protein 10% inclusion)
Rice	26.97	26.87	27.08
Oats	15.88	15.37	12.88
Wheat	14.45	14.39	14.50
Corn Gluten	14.45	14.23	14.24
Wheat Gluten	10.12	5.92	3.15
Novel fermented protein	0.0	5.0	10.00
Beet pulp	1.93	1.92	1.93
Brewer’s yeast	0.96	0.96	0.97
Calcium carbonate	1.45	1.44	1.45
Salt	0.96	0.96	0.97
Choline chloride 70%	0.16	0.12	0.15
Potassium chloride	0.63	0.62	0.59
DL-Methionine	0.14	0.14	0.15
L-Lysine	0.00	0.38	0.23
Naturox (Kemin)	0.1	0.10	0.1
Monocalcium phosphate	1.11	0.96	0.97
Vitamin & Mineral Premix	0.29	0.29	0.29
MCD	0.40	0.40	0.40
Potassium sorbate	0.20	0.20	0.20
Termox Dry (Kemin)	0.15	0.15	0.15
Toxin Binder	0.20	0.20	0.20
Sodium Benzoate	0.10	0.10	0.10
Termox Liquid (Kemin)	0.03	0.03	0.03
Fish oil	2.41	2.40	2.42
Sunflower Oil	5.95	5.88	5.90
Flaxseed Oil	0.96	0.96	0.97
Total	100	100	100

### Feeding trial

The feeding trial was conducted at Invetus, the largest Australasian veterinary contract research organization under the Institutional Animal Care and Use Committee protocol, authorised with the trial number RIU C 22179 W.

Seven healthy adult beagles with an average body weight of around 12 to 20 kg were individually housed in pens. The dogs received two feedings per day at time with water *ad libitum*.

### Study design

A total of seven dogs were enrolled into this study of which 5 dogs were fed with two different trial diets (3 dogs were fed with 5% fermented protein and another 2 dogs were fed with 10% fermented protein formulation) and 2 dogs were fed a control diet that contains 0% fermented protein and served as negative control throughout the conduct of this study for a period of 21 days.

From day 1–4 the 5 trial dogs and 2 control dogs were fed with a transition diet that consists of 75% standard diet (Cobbers Working dog kibble routinely fed at WRC) and 25% new diet. On days 5–8, dogs were fed with a transition diet that consisted of 50% standard diet and 50% new diet and on days 9–12, dogs were fed with a transition diet that consisted of 25% standard diet and 75% new diet. Following these 12 days of transition feeding, the 5 trial dogs were fed with 100% of the trial diet between days 13–20. The individual faecal samples were collected on day 1 and on day 21 for comparative microbiome analyses. The 2 control dogs received 100% control diet. Feed consumption and faecal scoring were recorded daily for all dogs. The diet schedule and the details of the animals are mentioned in Tables 2, 3. Tash and Buk were fed with control diets whereas Annie, Queenie, Kale, Huxley, Jasmine were fed with the test diets by replacing the basal diet with the fermented protein as mentioned in the Table 2.

**Table 2:** Diet schedule of the fermented protein (test) study in dogs.

Days	Diet schedule
Days 1–4	5 trial dogs (3 on 5% and 2 on 10% protein test diets) + 2 controls (0% protein diet); Housed dogs by group into three communal pens. Transition feeding with 75% (262.5 g) standard diet and 25% (87.5 g) new diet with fermented protein; Recorded food consumption and faecal scoring; Collected individual faecal samples into yellow-top jars and submerged in faecal storage solution. Jars were labelled with study number, dog ID and collection day and stored frozen at −20°C.
Days 5–8	5 trial dogs (3 on 5% and 2 on 10% protein test diets) + 2 controls (0% protein diet); Transition feeding with 50% (175 g) standard diet and 50% (175 g) new diet; Recorded food consumption and faecal scoring.
Days 9–12	5 trial dogs (3 on 5% and 2 on10% protein test diets) + 2 controls (0% protein diet); Transition feeding with 25% (87.5 g) standard diet and 75% (262.5 g) new diet; Recorded food consumption and faecal scoring.
Days 13–20	5 trial dogs (3 on 5% and 2 on10% protein test diets) + 2 controls (0% protein diet); New food 100% (350 g); Recorded food consumption and faecal scoring.
Day 21	Recorded food consumption and faecal scoring; Collected individual faecal samples into yellow-top jar and submerged in faecal storage solution. Jars labelled with Study number, Animal ID and collection date.

**Table 3:** Details of the test animals.

S. No.	Animal ID	Details
1	Tash 1347	Control
2	Buk 3850	Control
3	Annie 3610	Test 1 (5%fermented protein)
4	Queenie 4339	Test 1 (5% fermented protein)
5	Kale 5636	Test 1 (5% fermented protein)
6	Huxley 5632	Test 2 (10% fermented protein)
7	Jasmine 7812	Test 2 (10% fermented protein)

The nutrient composition of the fermented protein source and the experiment protein diets are presented in Tables 4, 5. The proximate composition of the fermented protein and the diets were analyzed by standard AOAC test methods.

**Table 4:** Nutrient composition of fermented protein.

S. No.	Description	Value	Test method
1.	Dry matter, %	94.4	By calculation; AOAC 930.15 (for moisture)
2.	Crude protein, %	72.2	AOAC 984.13
3.	Fat, %	5.3	AOAC 2003.06
4.	Crude Fiber, %	<1.0	AOAC 962.09
5.	Ash, %	7.7	IS 14827–2000
6.	Gross energy, MJ/kg	21.7	By calculation

**Table 5:** Nutrient composition of dry kibbles.

S. No.	Description	Control	Test 1 (fermented protein 5%)	Test 2 (fermented protein 10%)
1.	Moisture, %	7.5	7.5	7.5
2.	Crude protein, %	24.0	24.0	24.0
3.	Fat, %	10.0	10.0	10.0
4.	Crude Fiber, %	1.7	1.6	1.7
5.	Ash, %	5.2	5.7	5.8
6.	Gross energy, MJ/kg	14.6	14.6	14.5

### Faecal scoring and testing

Faecal scoring was done on a daily basis from day 1 to 21 and the scoring was done as per the Waltham faeces scoring system (24). The Waltham scale utilizes a scale of 1–5 with half numerical increments, covering a range of very hard (score 1) and dry to entirely liquid faeces (score 5) (24, 25). The mean Waltham score was calculated over the trial period to determine the overall stool consistency. The cut-off score for diarrhea was set at a mean score of 3.5. This also entails that control animals whose mean Waltham score exceeded this value were labelled as diarrhea-positive and vice versa. The faecal consistency scoring system is the most common stool scoring manual which can reflect intestinal health of the animals (26). Faecal condition scores can provide insights into how a diet is being digested (otherwise utilized) by an animal. The colour is also helpful in understanding the digestibility of animals. Low scores (unformed, loose, diarrhea, etc) may indicate digestive upset, malabsorption, and/or possible hydration issues. On the other end of the spectrum, hard stools may indicate a lack of appropriate fiber, a water balance issue, etc. The routine use of faecal scoring systems with animals can provide an invaluable tool to veterinarians and animal managers when there are any changes with condition, consumption, and/or overall health.

### Faecal DNA isolation and metagenome analysis/faecal DNA extraction and sequencing

DNA from the faecal samples was extracted using a Bioline Isolate Faecal DNA kit (Meridian, cat.no#BIO-52082). Primers were selected to amplify the V3–V4 region of 16S rRNA genes as these regions display the maximum discriminatory power demonstrating sufficient sequence diversity using the forward primer ACTCCTACGGGAGGCAGCAG and reverse primer GGACTACHVGGGTWTCTAAT. Sequencing was performed on an Illumina MiSeq platform using 2 × 300 bp paired-end sequencing. The microbiota of the dogs fed with 5 and 10% protein diets—namely Annie 3610, Queenie 4339, Kale 5636, Huxley 5632, and Jasmine 7812—was investigated on days 1 and 21. This analysis was conducted to assess changes in the microbiota of dogs fed diets containing the fermented bacterial Protein between day 1 (beginning of the study) versus day 21 (end of the study).

### Data analysis

Sequence data were trimmed with Trimmomatic (v 0.39) and then fastq files were analyzed using DADA2 in QIIME2 v2020.6 to denoise and produce Amplicon Sequence Variants (ASVs). ASVs were clustered at 99% identity using the VSEARCH plugin. Taxonomy was assigned using the SILVA database (v138). Obtaining feature table was further filtered (features that were present in only a single sample and features with a total abundance of less than 10). A total of 172,249 reads remained for the analysis, with an average of 17,224 reads per sample. The number of features remains after the data filtering step is 557. The downstream statistical microbial data analyses and visualizations were done using Microbiome Analyst (27). The community profiling was conducted using the R Phyloseq and Vegan packages. Principal coordinates analysis (PCoA) employing the Bray-Curtis Index and PERMANOVA was utilized to visualize the clustering of samples based on their phylum and genus-level compositional profiles. The identification of significant features was performed using single-factor statistical comparisons with a *p*-value cutoff of 0.05 and the *t*-test/ANOVA statistical method. The sequence data used for analysis is available in NCBI under BioProject accession Number PRJNA1116051.

## Results

### Feed consumption and faecal score

**Figure 1:**
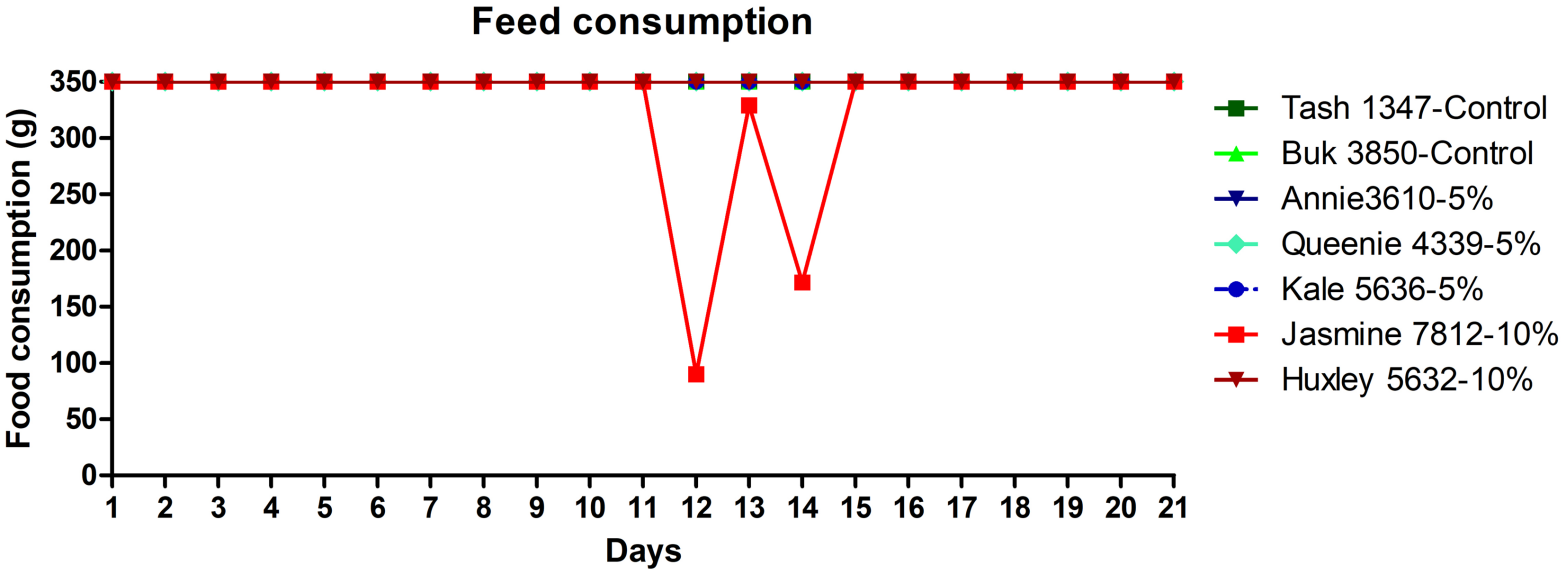
Feed consumption data of the dogs fed with the microbial fermented protein along with the control dogs. The line profiles demonstrate the feed consumption (in grams) of dogs over a period of 21 days. The feed consumption data points of the control dogs (Tash1347 and Buk3850) are marked in dark and light green colour respectively, 5% test protein fed dogs (Annie3610, Queenie4339 and Kale5636) are marked in dark blue, cyan and navy blue colours respectively, 10% test protein fed dogs (Jasmine7812 and Huxley5632) are marked in bright red and brown colour, respectively. The feed consumption of Jasmine7812 shows a dip on both 12th and 14th day.

**Figure 2:**
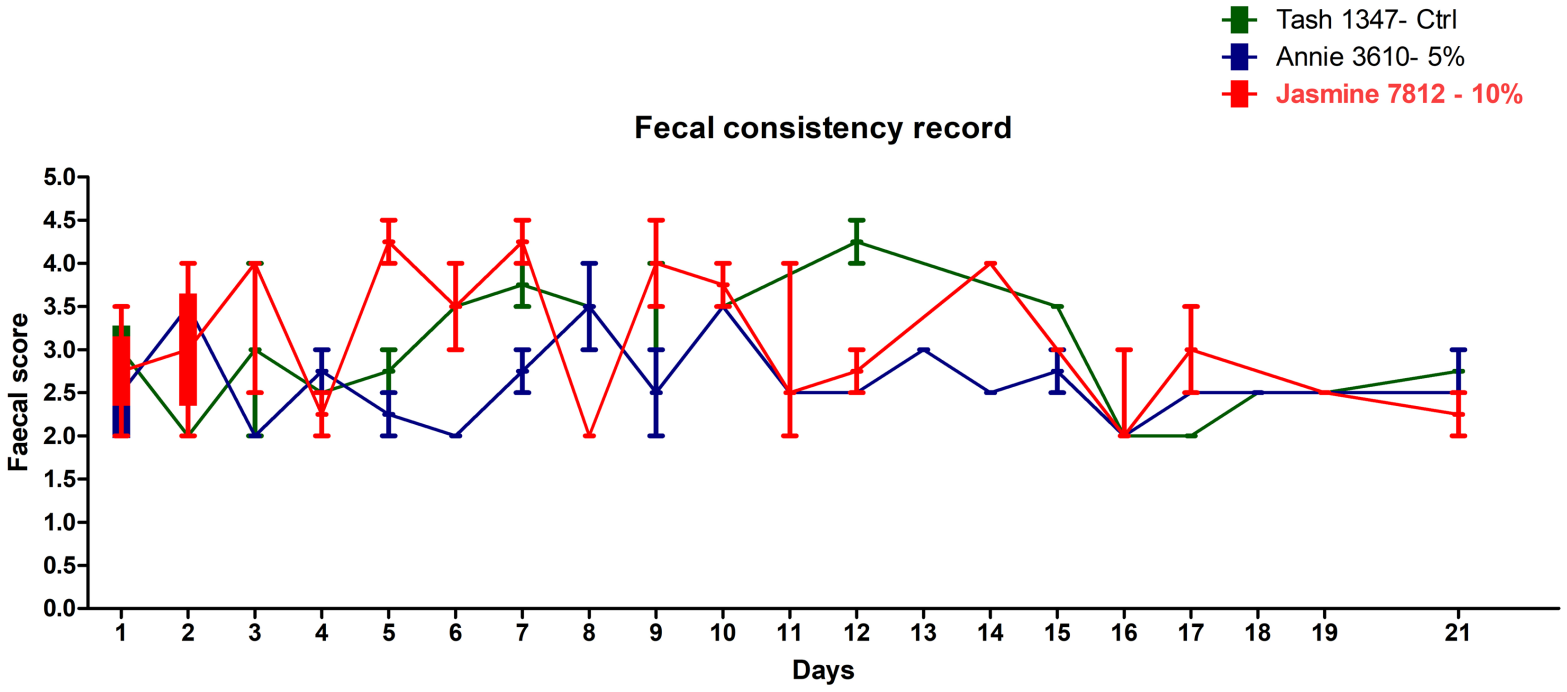
Representative faecal score of the dogs fed with the control (Tash1347, marked in green line) and fermented protein with 5% inclusion (Annie3610 marked in blue line) and 10% inclusion (Jasmine 7,812 marked in red line).

All the trial dogs were fed with 350 g/day as per calorific requirements. The feed consumption of the dogs fed with the fermented protein at 5 and 10% inclusions appear to be comparable to the control, demonstrating that the product is palatable and accepted by the dogs. There was a slight discrepancy in the feed consumption observed in one dog (Jasmine 7,812), which was fed with 10% fermented protein on days 12 and 14, however it became comparable to the control at the end of the study on day 21 (Figure 1). As part of this pilot study, we did not consider weight gain as the prime metric. However, we do not see a drastic change between the weights before and after the study (Table 6) with an exception in one of the animals, Jasmine 7,812 fed with 10% of the fermented protein did show lesser feed intake in the transition period between day 12 and day 14 which is also observed in the faecal consistency record (Figure 2).

**Table 6:** Weight of the animals before and after the study.

Dog ID and group	Initial weight (Kg)	Final weight (Kg)
	Day 1	Day 21
Tash 1347 (Control −0% test protein)	20.0	20.0
Buk 3850 (Control – 0% test protein)	14.7	15.0
Annie 3610 (5% test protein)	13.1	14.5
Queenie 4339 (5% test protein)	15.2	14.5
Kale 5636 (5% test protein)	15.0	15.0
Huxley 5632 (10% test protein)	13.7	14.0
Jasmine 7812 (10% test protein)	15.8	15.9

### Faecal scores

Faeces obtained from 0% protein control dog typically had a yellowish colouring to some parts of most faeces. 5% test protein faeces typically had an orangish colouring to some parts of some faeces. 10% test protein faeces had no major noticable differences to the colouring of faeces during the study. The aroma of faeces was different between the start of the study and the completion of the study for all groups. Clinical observations suggest that the diet containing fermented protein is acceptable by dogs throughout the study and hence reflects no palatability issues in the diets.

### Microbiome analyses

The microbiota of faecal samples from dogs fed with test protein diet and control diets was examined on days 1 and 21, respectively. The relative abundance, alpha and beta diversity were analyzed from the microbiome data.

### Abundance profiling

**Figure 3:**
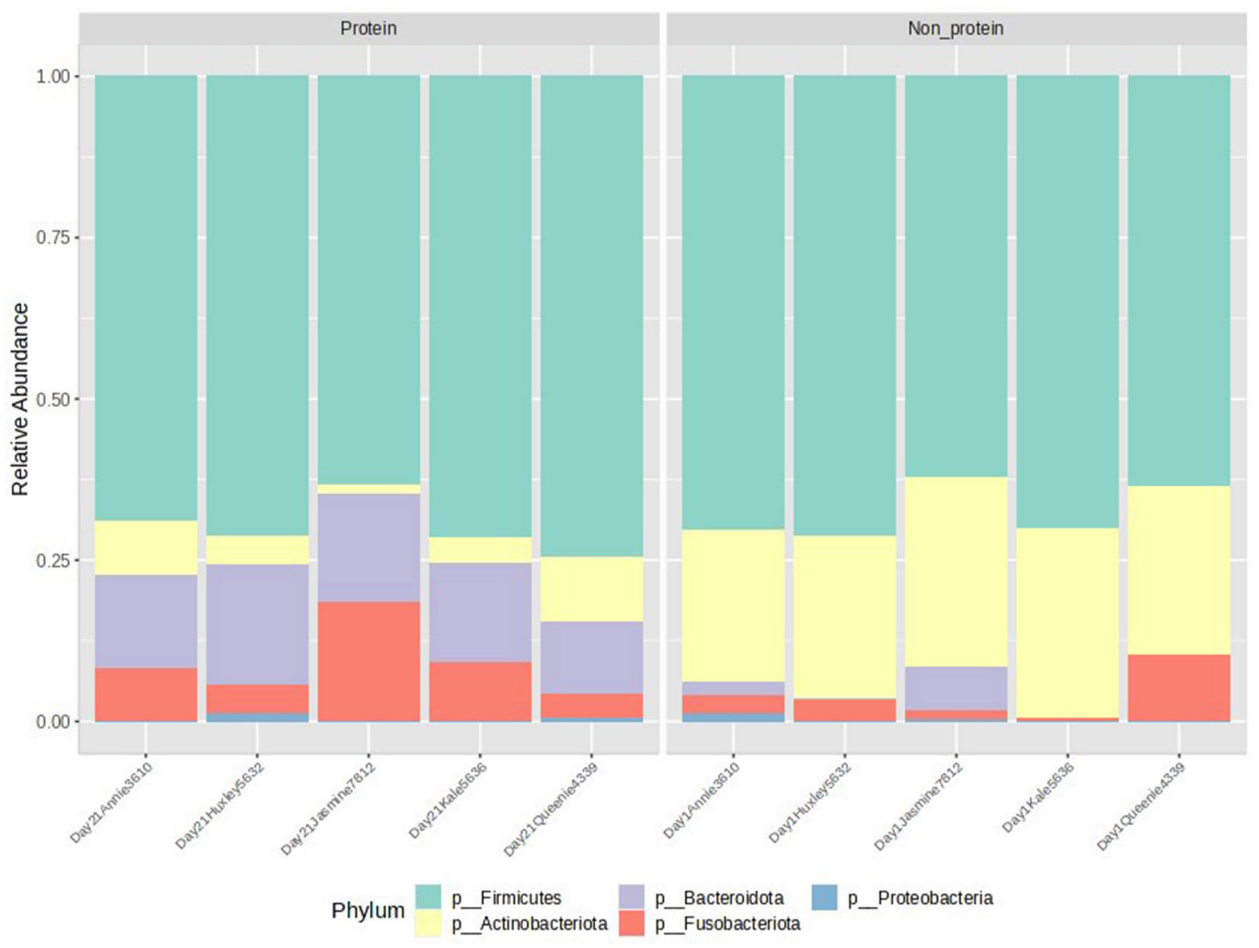
Relative abundance of the two groups. Members of the phyla Firmicutes, Actinobacteriota, Bacterioidota, Fusobacteriota, and Proteobacteria were observed. Protein group indicates animals fed with fermented protein (Day 21) and the non-protein group indicates animals fed with control diets (Day 1).

**Figure 4:**
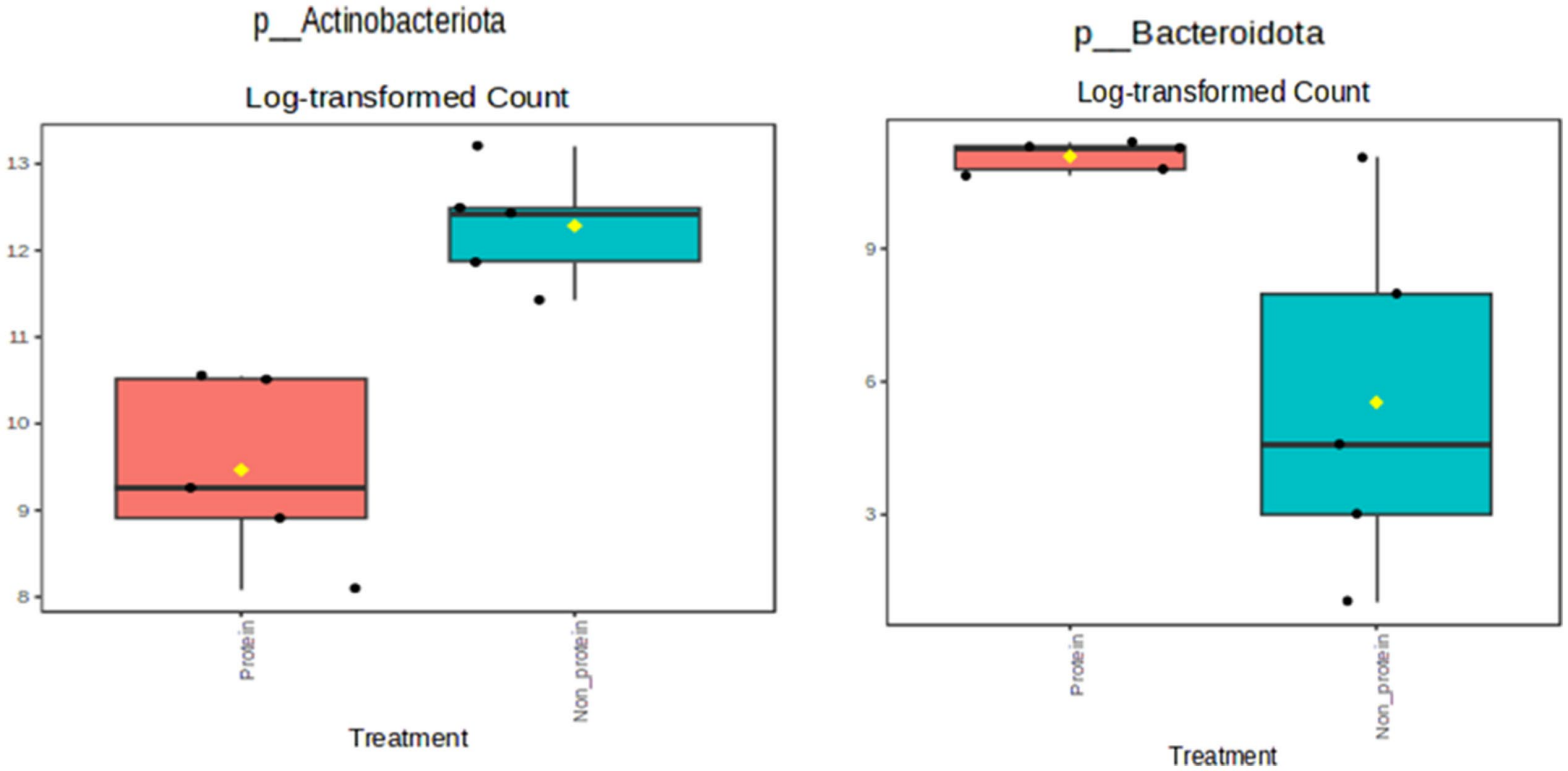
Single-factor statistical comparisons demonstrated a significant decrease in the phylum Actinobacteriota and an increase in the phylum Bacteroidota in the protein diet group (*p* < 0.05). Protein group indicates animals fed with fermented protein (Day 21) and the non-protein group indicates animals fed with control diets (Day 1).

The relative abundance at the phylum level is presented in Figure 3. Members of the Actinobacteriota phylum were reduced in the test protein group, while the Bacteroidota phylum showed an increased relative abundance (presented in yellow and purple, respectively, in Figure 3). Further statistical comparisons between the two groups demonstrated that these differences are significant (Figure 4). Firmicutes was the most abundant phylum detected in both the groups. The terms non-protein and protein corresponds to day 1 and day 21 diets fed to the animals.

**Figure 5:**
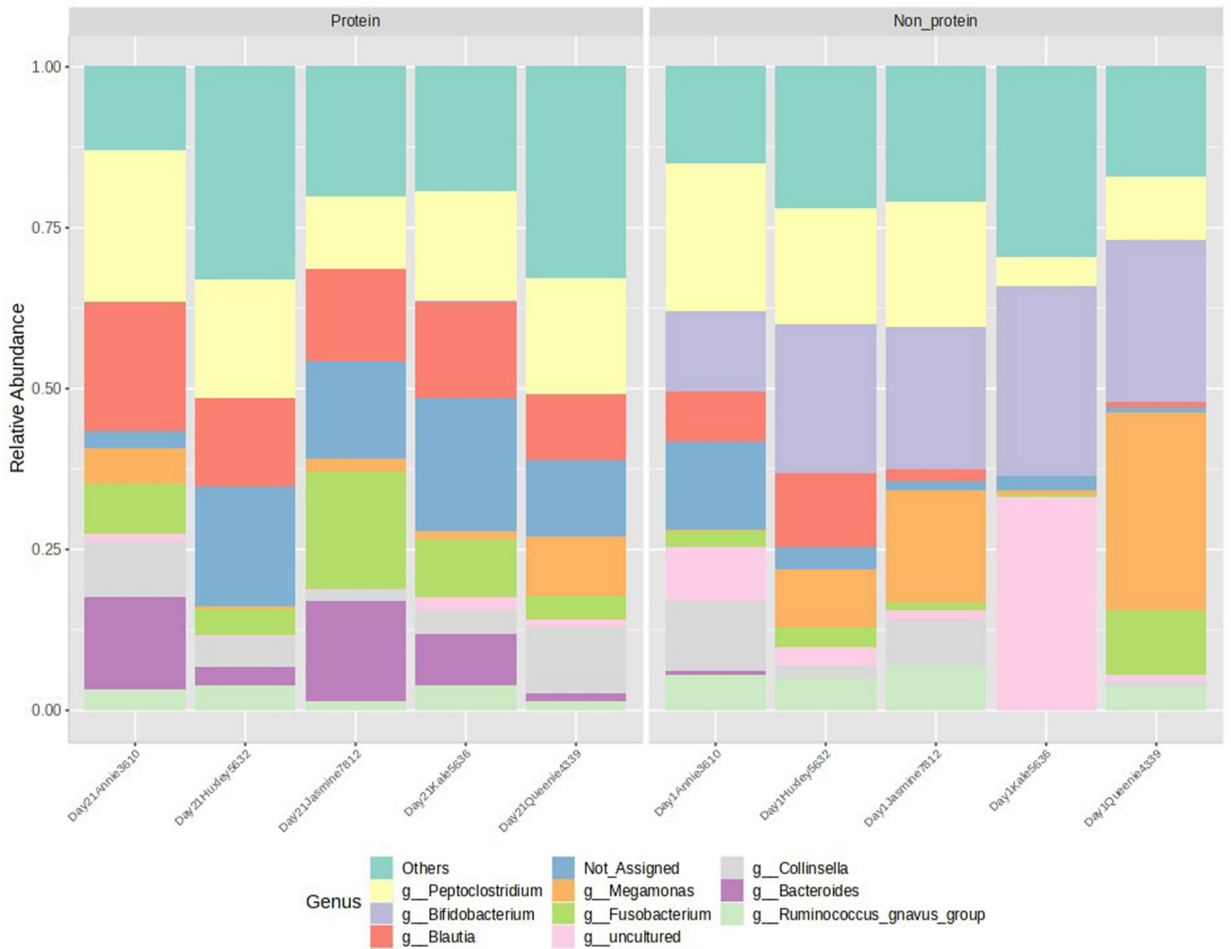
Relative abundance of top 10 genera detected in the two groups. Peptoclostridium, Bifidobacterium, Blautia, Megamonas, Fusobacterium, Collinsella, Bacteroides, Ruminococcus group of bacteria were observed. Protein group indicates animals fed with fermented protein (Day 21) and the non-protein group indicates animals fed with control diets (Day 1).

The relative abundance of the top 10 genera, visualized in a stacked bar chart, shows that members of the *Bifidobacterium* genus (presented in light purple in Figure 5) has a reduced relative abundance in the protein diet group compared to the non-protein diet group, while members of the *Bacteroides* and *Fusobacterium* genera (presented in dark purple and dark green, respectively, in Figure 5) show an increase. However, statistical comparison between the two groups reveals that these differences are not significant (with a *p*-value cutoff of 0.05).

### Single-factor statistical comparison between the test group studies

Single-factor statistical comparisons were used to determine if there were significant differences in the abundance of specific features between these groups. The results showed a significant reduction in the phylum Actinobacteriota (*p* < 0.05), but an increase in the phylum Bacteroidota in the protein diet group (Figure 4). No significant features were identified at the genus level with a *p*-value cutoff of 0.05.

### Alpha and beta diversity profiling

No significant difference was found in the Shannon and Simpson alpha diversity values, along with the Chao1 richness index, between the two groups, at the genus and phylum levels (*p* > 0.05).

**Figure 6:**
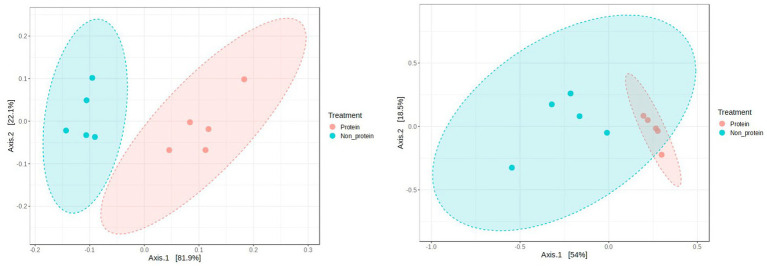
Principal coordinates analysis (PCoA) of gut microbiota composition in the two groups (left panel: at phylum level, right panel: at genus level). Distance method: Bray-Curtis index, Statistical method PERMANOVA’. *p*-value = 0.015. Protein group indicates animals fed with fermented protein (Day 21) and the non-protein group indicates animals fed with control diets (Day 1).

Principal Coordinate Analysis (PCoA) was employed to explore and visualize similarities and dissimilarities in the overall microbiota compositions of the two groups. A statistically significant difference in beta diversity between two groups suggests distinctions in the composition of the communities within them (*p* = 0.015 at both phylum and genus level) (Figure 6). One of the leading indicators of a healthy gut microbiome is the increased richness and diversity of microorganisms (28). Dogs with gastro intestinal disorders have been reported to have lower diversity when compared to healthy dogs (29–32). Studies with corn fermented protein demonstrated good preservation of alpha and beta diversity (33). Hence, difference in beta diversity observed in the current study supports good intestinal health in the dogs fed with the fermented protein diets.

As canine health is influenced by diet and the gastrointestinal microbiome in terms of nutrient digestion and absorption (34), leveraging alternative, novel ingredients in the dietary supplementation of the pet foods without impacting their gut health is a significant factor. The gut microbiome harboring diverse bacteria is a cardinal immune and metabolic organ and evaluation of the faecal microbiome is the most accessible sample type for testing. Several lines of evidence has demonstrated imbalances in the intestinal microflora is related to diseases like inflammatory bowel disease, irritable bowel syndrome, obesity and diabetes in humans and animal models (35–39) and are corroborated with the diagnosis and determining dysbiosis indices (40–43). Our current study demonstrates acceptance of the novel diet in the pet foods albeit the small sample size.

## Discussion

Our observations on feed acceptance, overall health and faecal microbiome profiling in dogs fed diets containing proteins derived from greenhouse gases show that these proteins are well tolerated, without any digestive problems and side effects. The feed consumption and the faecal scores also support the acceptance of the fermented protein of bacterial origin in diets in dogs. Additionally, the microbiome profiling indicates no significant alpha diversity changes, indicating that the protein diet does not affect the overall richness and evenness of microbial community.

In terms of acceptance, throughout the study, both 5 and 10% protein mix were completely consumed by all the dogs indicating the smell and taste aspects of these sustainable protein fractions in the kibble mix were well accepted. This is in line with the earlier studies relating to acceptance of dry pet foods in dogs (44, 45). According to AAFCO recommendation, the minimum dietary protein requirement for a growing dog is 18% dry matter (DM) and 8% for an adult dog. Intake of fermented protein in the extruded products/kibbles can be considered as a good sign in the canine diets. Product appearance is one of the key characteristics in dry dog foods, as was found by Di Donfrancesco et al. (44) wherein the authors found that dry dog food kibble that is too light or too dark in appearance may receive lower overall liking scores from potential product purchasers. The colour of test diet kibbles used in the study were of light brown colour. In terms of weight changes, we observed that only a slight change in weight was observed in the dogs fed with both 5 and 10% protein diet. The weight changes were not expected for a short study duration kibble acceptance study (46).

The clinical observations indicated that both the diets containing 5 or 10% of the fermented protein did not cause any impact with respect to general alertness, water consumption and social behavior providing evidence that this alternative protein source is safe. Further, digestive health as measured through faecal colouring and consistency scoring also was supportive of a well-tolerated protein fraction in the diet, without any alterations to the stool formation. The elevated fiber levels in diets containing Torula Yeast and legume proteins reduced dry matter and organic matter digestibility and have shown lower apparent fat digestibility (47). Studies demonstrate that on comparing soybean meal to poultry byproduct meal in extruded dog diets, soybean meal tended to reduce the digestibility coefficients of dry matter, organic matter, acid hydrolysed fat, and gross energy (48) reported that the inclusion of a soluble yeast cell wall reduced the coefficient of fat digestibility in an extruded dog diet without affecting any other nutrient digestibility (49). In the current study, there were no issues observed in terms of digestibility with the fermented protein fed diets in dogs.

The potential impact of the protein diet on the faecal microbiota was investigated in dogs fed the protein diet, with day 1 representing the non-protein diet and day 21 representing the protein diet. Due to the limited number of dogs enrolled in the study, those fed both 5 and 10% protein diets were grouped together. There was no significant difference in alpha diversity between the two groups when investigating the Shannon, Simpson and Chao1 indexes. However, beta diversity analysis indicated a significant difference (*p* = 0.015) in community structure between the protein and non-protein diet groups, with clear separation of the clustered samples from each group. As described previously (50–52) the diversity of the bacterial community did not change after supplementation with the tested protein source in healthy dogs. Only a few bacterial genera differed significantly in relative abundance between the experimental groups. The results are similar to the study done with insect-based diets (house crickets and mulberry silkworm pupae) tested in dogs (17).

A significant reduction of the phylum Actinobacteriota in the dogs with protein diet group was observed. The most well-known Actinobacteriota are *Bifidobacterium*, which are homo—or heterolactic fermentative. Higher abundance of Actinobacteria has been observed in adult obese dogs, probably due to their role in the production of energetic SCFAs (53). In contrast to what was observed in the Actinobacteriota phylum, the Bacteroidota phylum showed an increased relative abundance in the test protein diet group. The most abundant genera of this phylum are *Bacteroides* and *Prevotella* (54), which play significant roles in human and animal gastrointestinal tracts and are known to reduce intestinal oxygen levels and promote the growth of strict anaerobic bacteria (55). Although not significant, members of the Bacteroides genus had an increased abundance in the protein diet group.

Similarly, abundance profiling showed an increase in the *Fusobacterium* genus in the protein diet group, although this was not statistically significant. It is worth noting that unlike in the humans, the *Fusobacterium* genus is one of the three predominant phyla composing the gut microbiota in adult dogs, representing around 20% of the total relative abundance (56). This phylum is commonly observed in healthy dogs (57) and is found in higher abundance in dogs and cats than in humans (58). Due to their ability to degrade proteins into amino acids and peptides (59), it is assumed that Fusobacteria are key bacteria in the gut metabolism of carnivorous animals (60).

*Faecalibacterium* and *Ruminococcus* belong to the Firmicutes phylum, which is one of the top three most abundant phyla of the gut microbiota, with a high diversity of species. *Faecalibacterium* uses metabolites to produce butyrate, serving as energy for enterocytes providing anti-inflammatory protection (61) or limiting the colonization of pathogens, such as *Salmonella* (62). Firmicutes was also the most abundant phylum in both the protein and non-protein diet groups in this study. The genus of Catenibacterium, that is part of the Erysipelotrichaceae family (phylum Firmicutes), was found to be higher in dogs fed with homemade diet, while it was decreased in faecal samples of dogs fed a dry kibble diet. Dogs fed a raw meat diet had the lowest abundances (63). A similar pattern was observed in this study, where Catenibacterium had low abundance in the faecal samples of both groups and was not among the top 10 genera detected which corroborates with the earlier research studies demonstrating their lower prevalence in the kibble diets. Blautia spp. is shown to have potential probiotic properties and are found to be involved in gut health (64, 65). Our studies also demonstrate the presence of these bacterial species which indicates the role of fermented protein in providing good gut health. Earlier reports wherein the canine diets fed with corn-fermented protein demonstrated that the overall richness and diversity of the faecal microbiota were maintained when compared to the traditional ingredients such as brewer’s dried yeast and distiller’s dried grains with solubles (33). The microbiome diversity in the current pilot study indicates a similar trend with good acceptance of the gas derived fermented protein diet and hence good gut health.

Beneficial gut bacteria play a crucial part in the regulation of the canine immune system, which is important for the growth of the gastrointestinal physiological structure (66). Thus, it is important that canine diets provide important and balanced nutrients for both the host and the gut microbime (34). Pet owners play a significant role in determining the canine diets. Plant-based diets are popular choices for vegetarian dog owners and for those with special health concerns such as GI diseases and food allergies (67). On the other hand, some owners prefer meat-based which consists of organs, meat, and bones (50, 68, 69). Meat-based diet seems to be the preferred diet by dog owners because of the stereotype that plant-based foods are indigestible fillers with lower concentrations of nutritional compounds (70). However, several health concerns have been raised against strictly meat-based diets that are nutritionally imbalanced, contaminated with heavy metal and excessive chronic intake has been related to toxicities across many species, including dogs (71). Hence, commercial pet foods are provided as alternative diets but might not be of high quality raw material (2). Considering the above factors, augmenting the canine diets with green house derived fermented protein seems to be a sustainable, cost-effective, alternative without compromising the gut health and palatability of the animals. Hence, the current study would pave way for a functional, alternative protein alternative in the canine diets paving new dimensions into the dietary adaptations in pet foods in the future.

## Conclusion and future directions

Dietary protein sources in pet diets are largely sourced from animal byproducts and hence sustainability and societal changes become critical factors. The current study demonstrates that the microbial fermented protein source included in the canine diets is palatable and did not show any adverse effects on growth or welfare of the animals. Microbial fermented protein sources could be viable, sustainable solutions bringing lesser carbon footprint solution in the pet food sector without compromising on the quality of the nutritional profile. The microbiome changes with no major clinical symptoms is another positive sign in terms of overall digestive health of the animals.

Possible limitations of this study could be small sample size, and short study duration. It would have been beneficial to collect and analyze multiple faecal samples throughout the study for comparison. Also, an increase in the number of dogs enrolled in the study would provide more evidence of the potential impact of the protein diet on the faecal microbiota. Multicentered, large cohort studies coupled with biochemical, molecular and clinical parameters in future would help in validating and delineating the mechanisms of the fermented protein in the canine diets. For any study evaluating the impact of dietary intervention, there is concern regarding the study’s duration and if it is long enough for adaptation. However, Lin et al. (72) reported that the microbiome of dogs stabilized 6 days after dietary intervention, suggesting that the 21-day adaptation in the current study should have been sufficient. Of note, the methods in the current study are similar to those previously utilized to evaluate the impact of dietary intervention on the faecal microbiota of dogs. Extended, long term studies would help in understanding the mechanism of the fermented protein in canine diets and warrants further research to delineate the long-term potential health implications of this novel protein source in pet foods. Considering the sustainability factors such as land and water use, the fermented protein is perennial source which is unaffected by any of the climatic factors. Leveraging fermented protein used in this study helps in achieving carbon negative and climate positive outcomes which in deed would help in overall reduction of the carbon footprint which is the critical need of the hour. With ongoing advancements in biology, fermentation technology, and process engineering, the commercial viability and scalability of fermented proteins are steadily improving, paving the way for their integration into commercial pet diets.

## Data Availability

The sequence data used for analysis is available in NCBI under BioProject accession Number PRJNA1116051.

## References

[1] Fortune Business Insights. Pet food market size, growth, trends | Industry Analysis [2029]. (2022). Available at: https://www.fortunebusinessinsights.com/industry-reports/pet-food-market-100554 (accessed November 24, 2022).

[2] Fox MW. Not fit for a dog!: the truth about manufactured dog and cat food Linden Publishing (2012).

[3] AAFCO (Association of American Feed Control Officials) Inc. Official Publication, Oxford, IN; (2011). 13:9781884956836.

[4] Baker DH Czarnecki-Maulden GL. Comparative nutrition of cats and dogs. Annu Rev Nutr. (1991) 11:239–63. doi: 10.1146/annurev.nu.11.070191.001323 1892700

[5] Elliott KF Rand JS Fleeman LM Morton JM Litster AL Biourge VC. A diet lower in digestible carbohydrate results in lower postprandial glucose concentrations compared with a traditional canine diabetes diet and an adult maintenance diet in healthy dogs. Res Vet Sci. (2012) 93:288–95. doi: 10.1016/j.rvsc.2011.07.032, PMID: 21944832

[6] Boari A Aste G Rocconi F Dalessandri A Vita S. Glargine insulin and high-protein-low-carbohydrate diet in cats with diabetes mellitus. Vet Res Commun. (2008) 32:243–5. doi: 10.1007/s11259-008-9119-x 18685984

[7] Tôrres CL Hickenbottom SJ Rogers QR. Palatability affects the percentage of metabolizable energy as protein selected by adult beagles. J Nutr. (2003) 133:3516–22. doi: 10.1093/jn/133.11.3516, PMID: 14608067

[8] Weber M Bissot T Servet E Sergheraert R Biourge V German AJ. A high-protein, high-Fiber diet designed for weight loss improves satiety in dogs. J Vet Intern Med. (2007) 21:1203–8. doi: 10.1111/j.1939-1676.2007.tb01939.x 18196727

[9] German AJ Holden SL Bissot T Morris PJ Biourge V. A high protein high fibre diet improves weight loss in obese dogs. Vet J. (2010) 183:294–7. doi: 10.1016/j.tvjl.2008.12.004, PMID: 19138868

[10] Diez M Nguyen P Jeusette I Devois C Istasse L Biourge V. Weight loss in obese dogs: evaluation of a high-protein, low-carbohydrate diet. J Nutr. (2002) 132:1685S–7S. doi: 10.1093/jn/132.6.1685s, PMID: 12042493

[11] Blanchard G Nguyen P Gayet C Leriche I Siliart B Paragon B-M. Rapid weight loss with a high-protein low-energy diet allows the recovery of ideal body composition and insulin sensitivity in obese dogs. J Nutr. (2004) 134:2148S–50S. doi: 10.1093/jn/134.8.2148s, PMID: 15284423

[12] Reynolds AJ Reinhart GA Carey DP Simmerman DA Frank DA Kallfelz FA. Effect of protein intake during training on biochemical and performance variables in sled dogs. Am J Vet Res. (1999) 60:789–95. doi: 10.2460/ajvr.1999.60.07.789 10407468

[13] Moyad MA Robinson LE Zawada ET Kittelsrud JM Chen D-G Reeves SG. Effects of a modified yeast supplement on cold/flu symptoms. Urol Nurs (2008) 28: 50–55. Available at: https://pubmed.ncbi.nlm.nih.gov/18335698 (accessed July 24, 2024). 18335698

[14] Stercova E Kumprechtova D Auclair E Novakova J. Effects of live yeast dietary supplementation on nutrient digestibility and fecal microflora in beagle dogs. J Anim Sci. (2016) 94:2909–18. doi: 10.2527/jas.2016-0584 27482677

[15] Lin C-Y Alexander C Steelman AJ Warzecha CM de Godoy MRC Swanson KS. Effects of a *Saccharomyces cerevisiae* fermentation product on fecal characteristics, nutrient digestibility, fecal fermentative end-products, fecal microbial populations, immune function, and diet palatability in adult dogs 1. J Anim Sci. (2019) 97:1586–99. doi: 10.1093/jas/skz064, PMID: 30770927 PMC6447260

[16] Davenport GM Block SD Adolphe JL. Effects of extruded pet foods containing dried yeast (*Saccharomyces cerevisiae*) on palatability, nutrient digestibility, and fecal quality in dogs and cats. Transl Anim Sci. (2023) 7:txad107. doi: 10.1093/tas/txad107, PMID: 37745285 PMC10516452

[17] Areerat S Chundang P Lekcharoensuk C Patumcharoenpol P Kovitvadhi A. Insect-based diets (house crickets and mulberry silkworm pupae): a comparison of their effects on canine gut microbiota. Vet World. (2023) 16:1627–35. doi: 10.14202/vetworld.2023.1627-1635, PMID: 37766696 PMC10521172

[18] Tucci FJ Rosenzweig AC. Direct methane oxidation by copper-and Iron-dependent methane monooxygenases. Chem Rev. (2024) 124:1288–320. doi: 10.1021/acs.chemrev.3c00727, PMID: 38305159 PMC10923174

[19] Wang G Yang P Zhou X. Identification of the driving forces of climate change using the longest instrumental temperature record. Sci Rep. (2017) 7:46091. doi: 10.1038/srep46091, PMID: 28387247 PMC5384247

[20] Fei Q Guarnieri MT Tao L Laurens LML Dowe N Pienkos PT. Bioconversion of natural gas to liquid fuel: opportunities and challenges. Biotechnol Adv. (2014) 32:596–614. doi: 10.1016/j.biotechadv.2014.03.011, PMID: 24726715

[21] He L Lidstrom ME. Utilisation of low methane concentrations by methanotrophs. Adv Microb Physiol. (2024) 85:57–96. doi: 10.1016/bs.ampbs.2024.04.005, PMID: 39059823

[22] Clomburg JM Crumbley AM Gonzalez R. Industrial biomanufacturing: the future of chemical production. Science. (2017) 355:aag0804. doi: 10.1126/science.aag0804 28059717

[23] Subbian E Thillai K Duraiswami D Kumar VMLD Sairam N Shingala PG. Processes for fermentation and purification of value added products from gaseous substrates. (2017). Available at: https://patents.google.com/patent/EP3455341A4/en (accessed July 24, 2024).

[24] Moxham G. Waltham feces scoring system – a tool for veterinarians and pet owners: how does your pet rate? Waltham Focus. (2001) 11:24–5.

[25] Cavett CL Tonero M Marks SL Winston JA Gilor C Rudinsky AJ. Consistency of faecal scoring using two canine faecal scoring systems. J Small Anim Pract. (2021) 62:167–73. doi: 10.1111/jsap.13283 33491796

[26] German AJ Titcomb JM Holden SL Queau Y Morris PJ Biourge V. Cohort study of the success of controlled weight loss programs for obese dogs. J Vet Intern Med. (2015) 29:1547–55. doi: 10.1111/jvim.13629, PMID: 26426704 PMC4895666

[27] Dhariwal A Chong J Habib S King IL Agellon LB Xia J. Microbiome analyst: a web-based tool for comprehensive statistical, visual and meta-analysis of microbiome data. Nucleic Acids Res. (2017) 45:W180–8. doi: 10.1093/nar/gkx295, PMID: 28449106 PMC5570177

[28] Ziese A-L Suchodolski JS. Impact of changes in gastrointestinal microbiota in canine and feline digestive diseases. Vet Clin North Am Small Anim Pract. (2020) 51:155–69. doi: 10.1016/j.cvsm.2020.09.004, PMID: 33131916

[29] Suchodolski JS Markel ME Garcia-Mazcorro JF Unterer S Heilmann RM Dowd SE. The fecal microbiome in dogs with acute diarrhea and idiopathic inflammatory bowel disease. PLoS One. (2012) 7:e51907. doi: 10.1371/journal.pone.0051907 23300577 PMC3530590

[30] Isaiah A Parambeth JC Steiner JM Lidbury JA Suchodolski JS. The fecal microbiome of dogs with exocrine pancreatic insufficiency. Anaerobe. (2017) 45:50–8. doi: 10.1016/j.anaerobe.2017.02.010, PMID: 28223257

[31] Félix AP Souza CMM De Oliveira SG. Biomarkers of gastrointestinal functionality in dogs: a systematic review and meta-analysis. Anim Feed Sci Technol. (2021) 283:115183. doi: 10.1016/j.anifeedsci.2021.115183

[32] Díaz-Regañón D García-Sancho M Villaescusa A Sainz Á Agulla B Reyes-Prieto M. Characterization of the fecal and mucosa-associated microbiota in dogs with chronic inflammatory enteropathy. Animals. (2023) 13:326. doi: 10.3390/ani13030326, PMID: 36766216 PMC9913788

[33] Kilburn-Kappeler LR Doerksen T Lu A Palinski RM Lu N Aldrich CG. Comparison of the effect of corn-fermented protein and traditional ingredients on the fecal microbiota of dogs. Vet Sci. (2023) 10:553. doi: 10.3390/vetsci10090553, PMID: 37756074 PMC10536651

[34] Baritugo KA Bakhsh A Kim B Park S. Perspectives on functional foods for improvement of canine health and treatment of diseases. J Funct Foods. (2023) 109:105744. doi: 10.1016/j.jff.2023.105744

[35] Kelsen JR Wu GD. The gut microbiota, environment and diseases of modern society. Gut Microbes. (2012) 3:374–82. doi: 10.4161/gmic.21333, PMID: 22825455 PMC3463495

[36] Handl S German AJ Holden SL Dowd SE Steiner JM Heilmann RM. Faecal microbiota in lean and obese dogs. FEMS Microbiol Ecol. (2013) 84:332–43. doi: 10.1111/1574-6941.12067, PMID: 23301868

[37] Gevers D Kugathasan S Denson LA Vázquez-Baeza Y Van Treuren W Ren B. The treatment-naive microbiome in new-onset Crohn’s disease. Cell Host Microbe. (2014) 15:382–92. doi: 10.1016/j.chom.2014.02.005 24629344 PMC4059512

[38] Cox LM Blaser MJ. Antibiotics in early life and obesity. Nat Rev Endocrinol. (2014) 11:182–90. doi: 10.1038/nrendo.2014.210, PMID: 25488483 PMC4487629

[39] Guard BC Suchodolski JS. HORSE SPECIES SYMPOSIUM: canine intestinal microbiology and metagenomics: from phylogeny to function 1. J Anim Sci. (2016) 94:2247–61. doi: 10.2527/jas.2015-0029, PMID: 27285902

[40] Werner M Suchodolski JS Lidbury JA Steiner JM Hartmann K Unterer S. Diagnostic value of fecal cultures in dogs with chronic diarrhea. J Vet Intern Med. (2020) 35:199–208. doi: 10.1111/jvim.15982, PMID: 33277779 PMC7848338

[41] AlShawaqfeh M Wajid B Minamoto Y Markel M Lidbury J Steiner J. A dysbiosis index to assess microbial changes in fecal samples of dogs with chronic inflammatory enteropathy. FEMS Microbiol Ecol. (2017) 93:fix136. doi: 10.1093/femsec/fix136 29040443

[42] Honneffer JB Minamoto Y Suchodolski JS. Microbiota alterations in acute and chronic gastrointestinal inflammation of cats and dogs. World J Gastroenterol. (2014) 20:16489–97. doi: 10.3748/wjg.v20.i44.16489, PMID: 25469017 PMC4248192

[43] Suchodolski JS. Analysis of the gut microbiome in dogs and cats. Vet Clin Pathol. (2021) 50:6–17. doi: 10.1111/vcp.13031, PMID: 34514619 PMC9292158

[44] Di Donfrancesco B Koppel K Swaney-Stueve M Chambers E. Consumer acceptance of dry dog food variations. Animals. (2014) 4:313–30. doi: 10.3390/ani4020313 26480043 PMC4494379

[45] Di Donfrancesco B Koppel K Aldrich CG. Pet and owner acceptance of dry dog foods manufactured with sorghum and sorghum fractions. J Cereal Sci. (2018) 83:42–8. doi: 10.1016/j.jcs.2018.07.011

[46] Sagols E Hours MA Daniel I Feugier A Flanagan J German AJ. Comparison of the effects of different kibble shape on voluntary food intake and palatability of weight loss diets in pet dogs. Res Vet Sci. (2019) 124:375–82. doi: 10.1016/j.rvsc.2019.04.023, PMID: 31075615

[47] Holt DA Aldrich CG. Evaluation of Torula yeast as a protein source in extruded feline diets. J Anim Sci. (2022) 100:skac327. doi: 10.1093/jas/skac327, PMID: 36209420 PMC9733508

[48] Vanelli K de Oliveira ACF Sotomaior CS Weber SH Costa LB. Soybean meal and poultry offal meal effects on digestibility of adult dogs diets: systematic review. PLoS One. (2021) 16:e0249321. doi: 10.1371/journal.pone.0249321, PMID: 34043623 PMC8158863

[49] Stephanie TC Putarov TC Volpe LM Alberto C Beatriz M Carciofí AC. Effects of the solubility of yeast cell wall preparations on their potential prebiotic properties in dogs. PLoS One. (2019) 14:e0225659–9. doi: 10.1371/journal.pone.0225659, PMID: 31765439 PMC6878821

[50] Beloshapka AN Dowd SE Suchodolski JS Steiner JM Duclos L Swanson KS. Fecal microbial communities of healthy adult dogs fed raw meat-based diets with or without inulin or yeast cell wall extracts as assessed by 454 pyrosequencing. FEMS Microbiol Ecol. (2013) 84:532–41. doi: 10.1111/1574-6941.12081 23360519

[51] Gagné JW Wakshlag JJ Simpson KW Dowd SE Latchman S Brown DA. Effects of a synbiotic on fecal quality, short-chain fatty acid concentrations, and the microbiome of healthy sled dogs. BMC Vet Res. (2013) 9:246. doi: 10.1186/1746-6148-9-246, PMID: 24313995 PMC4029452

[52] Kim D-H Jeong D Kang I-B Lim H-W Cho Y Seo K-H. Modulation of the intestinal microbiota of dogs by kefir as a functional dairy product. J Dairy Sci. (2019) 102:3903–11. doi: 10.3168/jds.2018-15639 30827566

[53] You I Kim MJ. Comparison of gut microbiota of 96 healthy dogs by individual traits: breed, age, and body condition score. Animals. (2021) 11:2432. doi: 10.3390/ani11082432, PMID: 34438891 PMC8388711

[54] Hand D Wallis C Colyer A Penn CW. Pyrosequencing the canine Faecal microbiota: breadth and depth of biodiversity. PLoS One. (2013) 8:e53115. doi: 10.1371/journal.pone.0053115, PMID: 23382835 PMC3561364

[55] Wexler AG Goodman AL. An insider’s perspective: Bacteroides as a window into the microbiome. Nature. Microbiology. (2017) 2:1–11. doi: 10.1038/nmicrobiol.2017.26, PMID: 28440278 PMC5679392

[56] Suchodolski JS Camacho J JãM S. Analysis of bacterial diversity in the canine duodenum, jejunum, ileum, and colon by comparative 16S rRNA gene analysis. FEMS Microbiol Ecol. (2008) 66:567–78. doi: 10.1111/j.1574-6941.2008.00521.x 18557939

[57] Vázquez-Baeza Y Hyde ER Suchodolski JS Knight R. Dog and human inflammatory bowel disease rely on overlapping yet distinct dysbiosis networks. Nat Microbiol. (2016) 1:1–5. doi: 10.1038/nmicrobiol.2016.177 27694806

[58] Alessandri G Milani C Mancabelli L Mangifesta M Lugli GA Viappiani A. The impact of human-facilitated selection on the gut microbiota of domesticated mammals. FEMS Microbiol Ecol. (2019) 95:fiz121. doi: 10.1093/femsec/fiz121, PMID: 31344227

[59] Doron L Coppenhagen-Glazer S Ibrahim Y Eini A Naor R Rosen G. Identification and characterization of Fusolisin, the *Fusobacterium nucleatum* autotransporter serine protease. PLoS One. (2014) 9:e111329. doi: 10.1371/journal.pone.0111329, PMID: 25357190 PMC4214739

[60] Bermingham EN Maclean P Thomas DG Cave NJ Young W. Key bacterial families (Clostridiaceae, Erysipelotrichaceae and Bacteroidaceae) are related to the digestion of protein and energy in dogs. PeerJ. (2017) 5:e3019. doi: 10.7717/peerj.3019, PMID: 28265505 PMC5337088

[61] Handl S Dowd SE Garcia-Mazcorro JF Steiner JM Suchodolski JS. Massive parallel 16S rRNA gene pyrosequencing reveals highly diverse fecal bacterial and fungal communities in healthy dogs and cats. FEMS Microbiol Ecol. (2011) 76:301–10. doi: 10.1111/j.1574-6941.2011.01058.x, PMID: 21261668

[62] Rivera-Chávez F Zhang Lillian F Faber F Lopez Christopher A Byndloss Mariana X Olsan Erin E. Depletion of butyrate-producing Clostridia from the gut microbiota drives an aerobic luminal expansion of Salmonella. Cell Host Microbe. (2016) 19:443–54. doi: 10.1016/j.chom.2016.03.004, PMID: 27078066 PMC4832419

[63] Scarsella E Sandri M Monego SD Licastro D Stefanon B. Blood microbiome: a new marker of gut microbial population in dogs? Vet Sci. (2020) 7:198. doi: 10.3390/vetsci7040198, PMID: 33291629 PMC7761930

[64] Pilla R Suchodolski JS. The role of the canine gut microbiome and metabolome in health and gastrointestinal disease. Front Vet Sci. (2020) 6:498. doi: 10.3389/fvets.2019.00498, PMID: 31993446 PMC6971114

[65] Liu X Mao B Gu J Wu J Cui S Wang G. Blautia—a new functional genus with potential probiotic properties? Gut Microbes. (2021) 13:1–21. doi: 10.1080/19490976.2021.1875796, PMID: 33525961 PMC7872077

[66] Mondo E Marliani G Accorsi PA Cocchi M Di Leone A. Role of gut microbiota in dog and cat’s health and diseases. Open Vet J. (2019) 9:253–8. doi: 10.4314/ovj.v9i3.10, PMID: 31998619 PMC6794400

[67] Dodd SAS Cave NJ Adolphe JL Shoveller AK Verbrugghe A. Correction: plant-based (vegan) diets for pets: a survey of pet owner attitudes and feeding practices. PLoS One. (2022) 17:e0268982. doi: 10.1371/journal.pone.0268982, PMID: 35588426 PMC9119536

[68] Kim J An J-U Kim W Lee S Cho S. Differences in the gut microbiota of dogs (*Canis lupus familiaris*) fed a natural diet or a commercial feed revealed by the Illumina MiSeq platform. Gut Pathog. (2017) 9:68. doi: 10.1186/s13099-017-0218-5, PMID: 29201150 PMC5697093

[69] Sandri M Monego SD Conte G Sgorlon S Stefanon B. Raw meat based diet influences faecal microbiome and end products of fermentation in healthy dogs. BMC Vet Res. (2016) 13:65. doi: 10.1186/s12917-017-0981-z, PMID: 28245817 PMC5331737

[70] Beynen A. (2014). COLUMN: Unfair war on grain use in petfoods. All About Feed. Available at: https://www.allaboutfeed.net/animal-feed/feed-processing/column-unfair-war-on-grain-use-in-petfoods/.

[71] Kim H-T Loftus JP Mann S Wakshlag JJ. Evaluation of arsenic, cadmium, Lead and mercury contamination in over-the-counter available dry dog foods with different animal ingredients (red meat, poultry, and fish). Front Vet Sci. (2018) 5:264. doi: 10.3389/fvets.2018.00264, PMID: 30410919 PMC6209665

[72] Lin C-Y Jha AR Oba PM Yotis SM Shmalberg J Honaker RW. Longitudinal fecal microbiome and metabolite data demonstrate rapid shifts and subsequent stabilization after an abrupt dietary change in healthy adult dogs. Anim Microbiome. (2022) 4:46. doi: 10.1186/s42523-022-00194-9, PMID: 35915514 PMC9341101

[73] National Research Council (NRC). Nutrient requirements of dogs and cats. Washington, DC: National Academies Press (2006).

